# Impact of Playing Experience on the Relationship between Basketball Release Velocity and Muscle Contraction Strength in Mid- and Long-Distance Jump Shots

**DOI:** 10.5114/jhk/196544

**Published:** 2025-04-30

**Authors:** Ming Li, Youngsuk Kim, Bin Zhu, Ze Zhang, Sukwon Kim

**Affiliations:** 1 College of Education and Sports Sciences, Yangtze University, Jingzhou, China.; 2 Department of Physical Education, Jeonbuk National University, Jeonju, Republic of Korea.

**Keywords:** electromyography, wavelet analysis, vertical release velocity, horizontal release velocity, strength training

## Abstract

This study aimed to explore the impact of playing experience on vertical release velocity (VV) and horizontal release velocity (HV) during mid- and long-distance jump shots, and the relationships among VV, HV, and the contraction intensity levels of primary muscles. The study utilized a cross-sectional experimental design, with 30 participants completing three effective jump shots at distances of 4.8 m and 6.75 m. Data were collected using a 3D motion capture system and an electromyography (EMG) recording system, with velocity-time data extracted to determine VV and HV, and EMG data analyzed using wavelet analysis. A two-factor mixed ANOVA was used to compare differences in VV and HV under different conditions, and Spearman correlation analysis was employed to explore relationships among VV, HV, and the energy values of major muscle frequency bands. The results indicated that playing experience significantly improved VV (p < 0.001). At mid-range distances, experienced athletes' VV positively correlated with the low frequency band (r = 0.479, p = 0.048) and near frequency band (r = 0.539, p = 0.038) energy values of the triceps brachii (TB), as well as the high frequency band (r = 0.679, p = 0.005) and mid-high frequency band (r = 0.571, p = 0.026) energy values of the anterior deltoid (AD). At long-range distances, VV negatively correlated with the low frequency band energy values of the flexor carpi radialis (r = −0.5, p = 0.045), the high frequency band of TB (r = −0.564, p = 0.028), and the near frequency band of AD (r = −0.496, p = 0.046). These findings suggest that playing experience significantly alters VV during jump shots, and this alteration may be associated with the overall activity of the TB, the explosive activity of the AD, and the baseline activity of the rectus femoris.

## Introduction

Shooting, as a key and commonly used scoring method in basketball games, is widely regarded as an essential component of the technique (Button et al., 2003; Fan et al., 2024; Okazaki and Rodacki, 2012). Among all forms of shooting, the jump shot not only has the highest efficiency (Knudson, 1993), but also the widest application in games (Okazaki et al., 2015). Research indicates that nearly half of the scores in games are attributed to jump shots, and its high frequency of use makes it an indispensable scoring strategy in matches (Okazaki et al., 2015; Tang and Shung, 2005). Therefore, mastering jump shot skills is crucial for players, regardless of the role they play in the team.

In basketball games, athletes often shoot from various distances to expand offensive space and execute strategies (Miller and Bartlett, 1996), aiming for effective scoring (Okazaki and Rodacki, 2012). However, as the shooting distance increases, shooting accuracy tends to change (Li, 2021; Marques et al., 2023). This variation may stem from the nervous system's reorganization of joint motion patterns, a natural compensatory mechanism to accommodate longer shooting ranges (Okazaki and Rodacki, 2012). In practical game scenarios, distances of 4.8 m and 6.75 m represent common situations for mid- and long-distance jump shots (França et al., 2022; Nakano et al., 2020; Okazaki and Rodacki, 2012). Therefore, gaining a deep understanding of the contraction performance of major joint muscles after this reorganization during jump shots at these distances will help improve basketball players’ shooting performance.

Just as adjustments in shooting distance can affect basketball release performance, playing experience also influences shooting outcomes by altering the contraction intensity of major muscles during shooting. For instance, compared to novice athletes, experienced athletes exhibit longer activation times in their arm muscles during shooting, and this extended activation time is reflected in increased contraction intensity, which impacts shooting performance (Pakosz et al., 2021). Additionally, due to skill limitations, novice athletes often fail to generate sufficient muscle strength or achieve higher ball release heights, forcing them to rely on increasing segmental velocity to complete the shot, which in turn affects shooting performance (Hudson, 1985). Therefore, understanding the contraction intensity of the main propulsive muscles in experienced athletes can help improve shooting performance of novice athletes.

Previous research has extensively explored the effects of shooting distance and playing experience on shooting performance. Slegers et al. (2021) found that as shooting distance increased, the basketball’s release velocity significantly rose. Liu and Burton (1999) pointed out that with increasing distance, athletes might adjust the contraction intensity of major joint muscles to modify joint kinematics and meet the higher release velocity demands. Additionally, Hudson (1985) observed that experienced athletes displayed a greater release angle during shooting, indicating a greater reliance on vertical velocity output for successful shots. Pakosz (2011) further discovered that experienced athletes exhibited higher triceps brachii (TB) activation compared to novice athletes during shooting. Although those studies reveal the effects of release velocity and certain muscle activations, they lack detailed analysis of the relationship between directional velocities and muscle contraction intensity, as well as the specific connection between velocity and muscle contraction.

Given the critical impact of vertical and horizontal release velocities on the basketball's flight trajectory and the importance of muscle contraction performance in guiding strength training, understanding the relationship between muscle contraction intensity and release velocity is particularly important. Therefore, this study aimed to investigate the impact of playing experience on vertical release velocity (VV) and horizontal release velocity (HV) during mid- and long-distance jump shots, as well as the relationship among VV, HV, and the contraction intensities of primary muscles. We hypothesized that playing experience would enhance VV in basketball jump shots, and that increased jump shot distance would increase both VV and HV, likely due to the enhanced activity of key muscles during the shot.

## Methods

### Participants

This study recruited 30 male university basketball athletes, including 15 experienced team members (average age 22.3 ± 1.6 years; body height 185.8 ± 4.3 cm; body mass 78.7 ± 5.7 kg) with at least five years of competitive experience, and 15 novice athletes (average age 19.2 ± 0.6 years; body height 183.4 ± 2.6 cm; body mass 76.5 ± 5.1 kg) who had recently joined the team and had not yet participated in official competitions, but had mastered basic jump shot skills. To ensure the validity of the statistical analysis, the required sample size was estimated using G*Power 3.1.9.7 software, based on parameters α = 0.05, β = 0.8, and an effect size *f* = 0.31, which was calculated based on the observed data. This resulted in a minimum requirement of 24 participants. To further increase the statistical power, the sample size was increased to 30 participants. All participants were right-handed, had no neuromuscular diseases, and adhered to a regimen of basketball training 4 to 5 times per week, each session lasting 2.5 to 3.5 h. Before the study began, we thoroughly introduced the research methods, procedures, and potential risks to the participants, obtaining their written informed consent. This study was reviewed and approved by the Ethics Committee of the Jeonbuk National University (protocol code: JBNU2022-04-008-002; approval date: 26 May 2022), and it complied with the ethical standards of the Declaration of Helsinki.

### Measures

The jump shot motion capture was facilitated by a three-dimensional motion capture system, outfitted with thirteen infrared cameras (OptiTrack, LEYARD, Buffalo Grove, IL, USA), set to a sampling frequency of 240 Hz. Throughout this phase, reflective markers, 14 mm in diameter, were methodically affixed to fifty-seven critical anatomical landmarks on participants. Electromyography (EMG) data from the participants' right TB, flexor carpi radialis (FCR), anterior deltoid (AD), and rectus femoris (RF) were acquired using Trigno Avanti sensors (Delsys, Natick, MA, USA; dimension: 3.7 cm × 2.7 cm). These sensors operated at a sampling frequency of 1200 Hz and were each integrated with a bi-differential EMG sensor featuring silver bar electrodes (5 mm × 1 mm, inter-electrode distance 10 mm), securing a common-mode rejection ratio of 80 dB and an amplifier gain of 909. The designated areas were initially cleansed with sterile gauze and alcohol to eliminate any grease or debris, enhancing signal fidelity. This was followed by a gentle exfoliation of the skin using fine sandpaper to remove the stratum corneum, thereby lowering skin impedance. Conductive gel was then applied to the prepared area to further diminish electrical impedance at the electrode-skin interface. Finally, the electrodes were secured with sports tape to mitigate movement-induced artifacts. OptiTrack software (LEYARD, USA) was employed for the synchronous acquisition of kinematic and EMG data.

### Design and Procedures

Prior to commencing the experiment, staff comprehensively briefed participants on the procedural workflow. Subsequent to gaining a thorough understanding of the experiment's segments, participants performed a five-minute dynamic warm-up to prepare their bodies. To mitigate the warm-up's potential impact on shooting accuracy, a five-minute cooling period followed, intended to lower body temperature and facilitate oxygen consumption recovery (Bishop, 2003). Afterwards, participants were randomly allocated to either mid-range or long-range jump shooting tasks. During these tasks, participants were required to position themselves at predetermined spots and endeavor to replicate game-like shooting dynamics. Each distance demanded the completion of three successful jump shots (defined as the ball making it through the hoop), with a one-minute passive rest interposed between attempts at the same distance, and a three-minute passive rest implemented between attempts across different distances.

### Data Processing

Visual3D Setup x64 version 2023.10.1 (C-Motion, Inc., Germantown, MD, USA) was employed for extracting vertical and horizontal release velocity data of basketball shots, as well as for segmenting the phases of primary muscle EMG activity. The phases of muscle activity, specifically concerning the activities of the RF, AD, TB, and FCR, were delineated based on the distinct phases of knee, shoulder, elbow, and wrist joint motions. Specifically, the initial phase of knee joint motion referred to the moment the athlete began to squat, with the endpoint being the instant the feet left the ground. For the shoulder, elbow, and wrist joints, the onset of motion was defined as the moment during the standing release phase when the shoulder, elbow, and wrist began to extend (Okazaki et al., 2015), while the endpoint was marked by the instant of basketball release, which was determined by the moment the rigid body model of the basketball separated from the hand.

To facilitate the smoothing of data, velocity-time data were subjected to a 6-Hz bidirectional fourth-order Butterworth low-pass filter (Li et al., 2023). Under various experimental conditions, the collected velocity data were initially used for reliability assessment, subsequently selecting two datasets with the highest intraclass correlation coefficients (ICC greater than 0.8) for further analysis (Janicijevic et al., 2021; Li et al., 2024). Supported by R software version 4.3.2 (The R Foundation for Statistical Computing, Vienna, Austria), segmented raw EMG signals underwent processing through a 20–480-Hz band-pass filter, utilizing a fourth-order Butterworth filter for initial signal refinement. The subsequent signal denoising and decomposition were facilitated by the Maximum Overlap Discrete Wavelet Transform approach, with the optimization of the decomposition process achieved through the use of Daubechies-4 wavelets (Wei et al., 2012). This methodology delineated the EMG signal into four distinct frequency bands, comprising detail components labeled d1 through d4. These components sequentially represented the spectral characteristics of the original EMG signal across a spectrum from high to low frequency—d1 encapsulating high frequency, d2 mid-high frequency, d3 mid-low frequency, and d4 the low frequency range. Additionally, the a5 wave signified the approximate component at the fifth level of decomposition. For threshold estimation, a method predicated on the Median Absolute Deviation was employed (Jiang and Kuo, 2007). Subsequently, the band energy of the four primary muscles was computed, and the energy values obtained from two repeated calculations were averaged to complete the data analysis.

### Statistical Analysis

Statistical analysis of the data was conducted using R software version 4.3.2, with results presented as means ± standard deviations. The normality of data distribution was verified through the Shapiro-Wilk test. A two-factor mixed ANOVA (factors: group × distance) was employed to evaluate the differences between VV and HV, where *p* < 0.05 was considered statistically significant. The significance of differences was confirmed through post-hoc tests corrected by Bonferroni, to control for Type I errors. Effect size was calculated using Cohen's *d*, defined as: (Mean1 – Mean2) / SD_ pooled (Cohen, 1992). Given the limited representativeness and interpretability of d3 data, energy values for the d1, d2, d4, and a were selected for correlation analysis with VV and HV. This analysis employed the Spearman correlation method, setting r = 0.3 as the significance threshold, with *p* < 0.05.

## Results

### Release Velocity

Table 1 details the means and standard deviations for VV and HV. Descriptive statistics indicate that as the shooting distance increased to 6.75 m, for the novice group (NG), VV went up by 13.62%, and HV by 18.24%; for the experienced group (EG), VV increased by 16.34%, and HV by 11.5%. Statistical analysis showed significant main effects for the group (*p* < 0.001) and distance (*d* = 0.004) on VV, without a significant interaction (*p* = 0.317). At 4.8 m, the EG significantly outperformed the NG (*p* < 0.001, *d* = 1.64); at 6.75 m, and the EG remained significantly higher than the NG (*p* < 0.001, *d* = 2.25). Furthermore, in the NG, increasing the shooting distance to 6.75 m significantly raised VV (*p* < 0.001, *d* = 1.19); in the EG, an increase to 6.75 m also significantly elevated VV (*p* < 0.001, *d* = 1.96). For HV, significant main effects were noted for group (*p* = 0.018) and distance (*p* = 0.032), with no significant interaction (*d* = 0.543). At 4.8 m, the EG significantly surpassed the NG (*p* = 0.018, *d* = 0.88). Additionally, in the NG, an increase in shooting distance to 6.75 m significantly enhanced HV (*p* = 0.004, *d* = 0.85); in the EG, a similar increase significantly boosted HV (*p* = 0.032, *d* = 1.15).

**Table 1:** Vertical and horizontal release velocities in jump shots: comparing two distances and groups (mean ± SD).

			Jump Shot Distance (m)		
Variable	Group			4.8	6.75
VV (m•s^−1^)	NG		3.89 ± 0.37		4.42 ± 0.52†
	EG		4.59 ± 0.47*[1.64]		5.34 ± 0.27*†[2.25]
HV (m•s^−1^)	NG		2.96 ± 0.6		3.5 ± 0.68†
	EG		3.39 ± 0.36*[0.88]		3.78 ± 0.32†[0.53]

VV = vertical release velocity; HV = horizontal release velocity; NG = novice group; EG = experienced group; *: significantly (p < 0.05) different from the NG; †: significantly (p < 0.05) different from the 4.8 m; effect sizes between the EG and the NG are reported in square brackets

**Table 2:** The relationship between the various levels of energy values of major muscles and the vertical and horizontal release velocities at a shooting distance of 4.8 m (r).

	NG			EG	
Energy values	VV	HV		VV	HV
FCR-d1	0.382	−0.7*		0.236	0.164
FCR-d2	0.304	−0.689*		0.161	0.329
FCR-d4	0.275	−0.725*		0.057	0.314
FCR-a	0.279	−0.629*		−0.029	0.225
TB-d1	−0.325	0.264		0.329	0.243
TB-d2	−0.261	0.207		0.343	0.236
TB-d4	−0.232	0.182		0.479*	−0.026
TB-a	−0.364	0.014		0.539*	−0.143
AD-d1	−0.221	−0.132		0.679*	−0.536*
AD-d2	−0. 239	−0.168		0.571*	−0.539*
AD-d4	0.029	−0.354		0.032	0.2
AD-a	−0.004	−0.361		−0.007	0.093
RF-d1	−0.121	0.068		0.15	−0.593*
RF-d2	−0.154	0.096		0.032	−0.461*
RF-d4	−0.082	0.104		−0.229	−0.239
RF-a	0.071	0.011		−0.239	−0.082

FCR = flexor carpi radialis; TB = triceps brachii; AD = anterior deltoid; RF = rectus femoris; d1 = high frequency band; d2 = mid-high frequency band; d4 = low frequency band; a = approximation level; NG = novice group; EG = experienced group; VV = vertical release velocity; HV = horizontal release velocity; *: significant at p < 0.05

**Table 3:** The relationship between the various levels of energy values of major muscles and the vertical and horizontal release velocities at a shooting distance of 6.75 m (r).

	NG			EG	
Energy values	VV	HV		VV	HV
FCR-d1	0.507*	−0.486*		−0.211	0.25
FCR-d2	0.514*	−0.475*		−0.15	0.221
FCR-d4	0.35	−0.546*		−0.5*	−0.014
FCR-a	0.414	−0.361		−0.375	−0.161
TB-d1	−0.125	0.693*		−0.564*	0.589*
TB-d2	−0.104	0.725*		−0.364	0.468*
TB-d4	−0.043	0.493*		−0.211	0.396
TB-a	0.221	0.203		−0.229	0.443
AD-d1	0.464*	−0.221		0.054	0.029
AD-d2	0.443	−0.211		0.118	−0.007
AD-d4	0.4	−0.421		−0.3	0.236
AD-a	0.453	−0.486*		−0.496*	0.236
RF-d1	0.393	−0.089		−0.103	0.45
RF-d2	0.289	0.079		−0.096	0.454
RF-d4	0.4	−0.15		−0.143	0.539*
RF-a	0.643*	−0.329		−0.25	0.582*

FCR = flexor carpi radialis; TB = triceps brachii; AD = anterior deltoid; RF = rectus femoris; d1 = high frequency band; d2 = mid-high frequency band; d4 = low frequency band; a = approximation level; NG = novice group; EG = experienced group; VV = vertical release velocity; HV = horizontal release velocity; *: significant at p < 0.05

**Figure 1:**
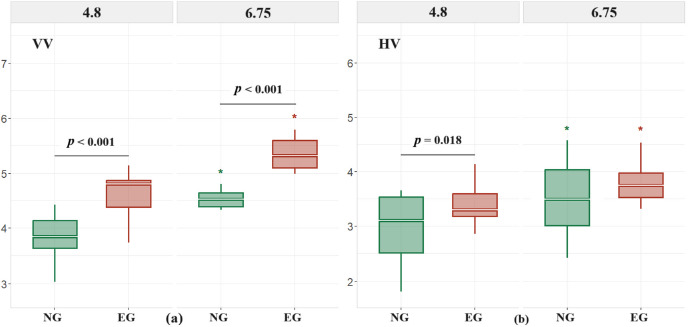
Vertical release velocity (VV) and horizontal release velocity (HV) for two groups during mid- and long-distance jump shots, divided into two panels: (a) VV, (b) HV. Each box plot in the figure contains the following features: Upper edge (Q3): Upper quartile; Upper whisker: Maximum value plus 1.5 times the interquartile range (IQR); Central white line: Median; Lower edge (Q1): Lower quartile; Lower whisker: Minimum value minus 1.5 times the IQR *Significant differences between different distances within groups are marked with asterisks; horizontal lines with corresponding p-values indicate significant differences between the novice group (NG) and the experienced group (EG)*.

### Mid-Range

In the NG, there was no significant correlation between the energy values of the four muscles and VV (*p* > 0.05). In relation to HV, only FCR's various frequency bands (d1, d2, d4, and a) exhibited a significant negative correlation (d1: r = −0.7, *p* = 0.004; d2: r = −0.689, *p* = 0.004; d4: r = −0.725, *p* = 0.002; a: r = −0.629, *p* = 0.012). In the EG, significant positive correlations were found between TB's d4 and a bands, and AD's d1 and d2 bands with VV (d4: r = 0.479, *p* = 0.048; a: r = 0.539, *p* = 0.038; d1: r = 0.679, *p* = 0.005; d2: r = 0.571, *p* = 0.026). In relation to HV, only AD's d1 and d2 bands, and RF's d1 and d2 bands showed a significant negative correlation (AD-d1: r = −0.536, *p* = 0.039; AD-d2: r = −0.539, *p* = 0.038; RF-d1: r = −0.593, *p* = 0.019; RF-d2: r = −0.461, *p* = 0.049).

### Long-Range

In the NG, significant positive correlations were found among FCR-d1, FCR-d2, DM-d1, and RF-a with VV (FCR-d1: r = 0.507, *p* = 0.044; d2: r = 0.514, *p* = 0.044; DM-d1: r = 0.464, *p* = 0.049; a: r = 0.643, *p* = 0.009). Regarding HV, FCR's d1, d2, and d4 bands and AD's a band exhibited significant negative correlations (d1: r = −0.486, *p* = 0.046; d2: r = −0.476, *p* = 0.047; d4: r = −0.546, *p* = 0.035; a: r = −0.486, *p* = 0.046); TB's d1, d2, and d4 bands showed significant positive correlations (d1: r = 0.693, *p* = 0.004; d2: r = 0.725, *p* = 0.002; d4: r = 0.493, *p* = 0.046). In the EG, FCR-d4, TB-d1, and AD-a exhibited significant negative correlations with VV (d4: r = −0.5, *p* = 0.045; d1: r = −0.564, *p* = 0.028; a: r = −0.496, *p* = 0.046). Regarding HV, only TB's d1 and d2 bands, along with RF's d4 and a bands demonstrated significant positive correlations (d1: r = 0.589, *p* = 0.021; d2: r = 0.468, *p* = 0.048; d4: r = 0.539, *p* = 0.038; a: r = 0.582, *p* = 0.023).

## Discussion

This study explored the effects of playing experience on VV and HV during mid- and long-range jump shots (4.8 m and 6.75 m), as well as the correlation among the energy values of primary muscle frequency bands, VV and HV. The main findings show that playing experience enhanced VV of the basketball during jump shots. With an increase in shooting distance, experienced athletes tended to perform their shots with increased VV, while novice athletes more often relied on increasing HV. Secondly, for mid-distance shots, novice athletes did not show a significant correlation between VV and major muscle activity, whereas HV negatively correlated with the energy values of all FCR frequency bands. Experienced athletes' VV positively correlated with TB's low and near frequency band energy values, as well as AD's high and mid-high frequency band energy values, while HV negatively correlated with AD and RF's high and mid-high frequency band energy values. For long-distance shots, novice athletes' VV positively correlated with the high and mid-high frequency bands of FCR, the high frequency band of AD, and the near frequency band energy values of RF, while HV negatively correlated with the high, mid-high, and low frequency band energy values of FCR. Experienced athletes' VV negatively correlated with FCR's low frequency band, TB's high frequency band, and AD's near frequency band energy values, while HV positively correlated with the high and mid-high frequency band energy values of TB and the low and near frequency band energy values of RF.

Regarding the finding that playing experience enhances VV of athletes during shooting, Hudson (1985) conducted a study on 22 college students with varying levels of basketball playing experience, observing differences in their shooting techniques. The results indicated that college athletes with extensive playing experience exhibited a greater release angle during shots. Considering the positive correlation between the release angle and vertical motion (Miller and Bartlett, 1993), our study's results are largely in agreement with Hudson's findings. Unlike Hudson's use of 2D video capture technology (Cine-Kodak Special camera), this research incorporated a more precise 3D motion capture system. This technological innovation enhanced data accuracy and ensured that the study's scientific validity and reliability were more thoroughly substantiated. Different impacts of shooting distance on athletes’ performance have been validated in previous research. Slegers et al. (2021) examined how shooting distance affected the jump shot performance of 12 experienced college athletes, finding that as shooting distance increased, the basketball's release velocity significantly rose, while the release angle remained unchanged. Given that experienced athletes exhibit a greater release angle (Hudson, 1985; Kambič et al., 2022), this implies that at longer shooting distances, they demonstrate higher VV, whereas novices show higher HV. Compared to previous work, this study, by recruiting more participants, enhanced statistical power and improved the representativeness and reliability of the research findings. Experienced athletes tend to adopt higher VV during jump shots, a strategy possibly aimed at optimizing the basketball's entry angle (Miller and Bartlett, 1993), thereby increasing the likelihood of scoring (Brancazio, 1981). Generally, a larger entry angle provides a greater margin for error (Brancazio, 1981), subsequently raising the probability of the ball successfully entering the hoop. Conversely, novice athletes tend to exhibit higher HV, possibly due to insufficient primary muscle strength, making it difficult to generate adequate vertical displacement of the ball to increase its flight time, hence they are more inclined to use a strategy that increases HV to ensure shooting success (Hudson, 1985).

In basketball, EMG studies primarily focus on the variations in muscle activation levels and activation timing among athletes of different skill levels. For instance, research by Pakosz (2011) indicated that during the shooting process, experienced athletes exhibited higher TB activation levels than novice athletes; another study found that experienced athletes had longer activation times for the TB during shooting (Pakosz et al., 2021). However, there is still a relative scarcity of research on the correlation between energy values of different muscle frequency bands and release velocities among athletes of varying experience levels.

This study is the first to uncover that during mid-distance shooting, VV of the basketball at release by experienced athletes is positively associated with the rapid contraction intensity of the AD, as well as the slow contraction strength and baseline activity intensity of the TB, while HV is negatively associated with the rapid and mid-rapid contraction intensities of the AD and the RF. Novice athletes did not show such correlations. This may be due to the AD's role in flexing the shoulder joint (Williams, 1949), which is related to a higher release angle (Knudson, 1993). Hence, the rapid contraction intensity of the AD is positively related to VV and negatively to HV. Given that elbow extension has been recognized as a strategy to increase release height (Knudson, 1993), along with the elbow joint's critical role in determining the release velocity of the basketball (Button et al., 2003), the findings that the slow contraction strength and baseline activity intensity of the TB are positively related to VV align with the observed performance of experienced athletes in our study. Moreover, considering that in mid-distance shots, the jumping phase tends to be more vertical (Okazaki et al., 2015), the rapid contraction intensity of the RF, which governs the motion of the knee joint, exhibits a negative correlation with HV. No such correlations were shown by novice athletes. We may speculate that this is because novice athletes, as new team members who have not been rigorously assessed, still maintain their inherent habits and movement patterns, leading to significant individual variations, and thus do not exhibit a consistent correlation pattern.

Another key finding of this study is that during long-distance shooting, novice athletes' VV positively correlated with the mid-rapid and rapid contraction intensities of the FCR, the rapid contraction intensity of the AD, and the baseline activity intensity of the RF. Conversely, HV negatively correlated with FCR's mid-rapid, rapid, and slow contraction intensities. For experienced athletes, VV negatively correlated with FCR's slow contraction intensity, TB's rapid contraction intensity, and AD's baseline activity intensity; HV positively correlated with TB's mid-rapid and rapid contraction intensities, as well as RF's slow contraction intensity and baseline activity intensity. The differing results at the two distances may be due to the need for basketball players to reorganize the coordinated movements of various body parts (Miller and Bartlett, 1996, 1993) as shooting distance increases, generating greater ball velocity to meet new task demands (Okazaki and Rodacki, 2012). Therefore, at long distances, novice and experienced athletes exhibit different outcomes compared to close-range shooting. We may speculate that the reason novice athletes show correlations in VV is that the rapid contraction behavior of the FCR leads to the wrist joint reaching peak angular velocity early in the flexion phase (Okazaki et al., 2006), causing the basketball to be released early during wrist flexion, thus making the shot contribute more to the vertical direction, and thereby showing a positive correlation with VV and a negative correlation with HV. Meanwhile, the shoulder flexion and knee extension movements dominated by the AD and the RF, respectively, both impact the vertical direction (Knudson, 1993; Okazaki et al., 2015; Williams, 1949), hence they both exhibit a positive correlation with VV.

The correlations observed in VV among experienced athletes are speculated to be due to the dominance of FCR's slow contraction in wrist joint activities. Unlike rapid contractions, slow contractions lead to the wrist joint reaching peak angular velocity only in the later stages of flexion (Okazaki et al., 2006), causing a deviation in the wrist's contribution to the vertical direction. With the increase in shooting distance, experienced athletes tend to show higher jump heights and smaller shoulder flexion angles (Elliott and White, 1989; Miller and Bartlett, 1993). This is because the ball is released before reaching the apex of the basketball jump shot (Vencúrik et al., 2021). To adapt to a smaller release angle, the release velocity is increased by accelerating elbow joint motion (Miller and Bartlett, 1996, 1993). Therefore, TB's rapid contraction intensity and AD's baseline activity intensity are negatively correlated with VV. The correlations with HV may be due to the need for faster elbow joint velocity to meet new shooting requirements as the shooting distance increases (Miller and Bartlett, 1993), hence TB's mid-rapid and rapid contraction intensities show a positive correlation with HV. Additionally, as distance increases, the lower limb movement during the jump prompts the center of gravity to move towards the basket (Elliott and White, 1989; Miller and Bartlett, 1993; Okazaki and Rodacki, 2012) to meet the demands of long-distance shooting. Considering that RF's slow and baseline activities dominate the entire extension process of the knee joint, this also leads to a positive correlation between RF's slow contraction and baseline activity intensities with HV.

The limitations of this study highlight potential directions for future research, encompassing three primary areas for exploration and improvement. First, the cross-sectional design adopted in this study, along with its reliance on correlational analysis methods, although facilitating the exploration of associations between variables, does not establish definitive causal relationships. Future research should consider employing longitudinal designs to establish causal links. Secondly, all participants in this study were male athletes, which limits the generalizability of the findings to female athletes. Future studies should include both male and female participants to expand the applicability of the research. Lastly, the data on athletes' shooting actions in this study were solely derived from a laboratory setting, which ensured a high degree of accuracy in technical data. However, this setting did not fully account for the complex factors that may influence technical performance in actual competition scenarios. Future research should adopt more advanced techniques to collect data in real-game environments, providing a more comprehensive understanding of how athletes of varying experience levels perform under realistic conditions.

## Conclusions

This study explored the impact of playing experience on mid- and long-distance jump shots. The findings reveal that playing experience significantly enhanced the basketball's VV. As the shooting distance increased, experienced athletes tended to complete their shots by increasing VV, while novice athletes were more inclined to increase HV. This difference may be associated with the overall activity of the TB, the explosive activity of the AD, and the baseline activity of the RF. Therefore, novice athletes should focus on strategically increasing their vertical velocity output during jump shot training. In strength training, attention should be given to comprehensive training of the TB (including explosive power and endurance), explosive strength training of the AD, and endurance and control training of the RF. These adjustments will help extend the basketball's flight time, increase the entry angle into the basket, and improve scoring opportunities.
